# Traumatic injury causes selective degeneration and TDP-43 mislocalization in human iPSC-derived *C9orf72*-associated ALS/FTD motor neurons

**DOI:** 10.1101/2024.03.21.586073

**Published:** 2024-03-26

**Authors:** Eric J. Martin, Citlally Santacruz, Angela Mitevska, Ian E. Jones, Gopinath Krishnan, Fen-Biao Gao, John D. Finan, Evangelos Kiskinis

**Affiliations:** 1 The Ken & Ruth Davee Department of Neurology, Feinberg School of Medicine, Northwestern University, Chicago, IL 60611, USA.; 2 Department of Mechanical and Industrial Engineering, University of Illinois at Chicago, Chicago, IL, USA.; 3 RNA Therapeutics Institute, University of Massachusetts Medical School, Worcester, MA 01605, USA.; 4 Department of Neurology, University of Massachusetts Medical School, Worcester, MA 01605, USA.; 5 Simpson Querrey Institute, Northwestern University, Chicago, Illinois 60611, USA.; 6 Department of Neuroscience, Northwestern University Feinberg School of Medicine, Chicago, IL, 60611, USA.

**Keywords:** Neurotrauma, amyotrophic lateral sclerosis (ALS), C9orf72, iPSC models, TDP-43, frontotemporal dementia (FTD), motor neurons, traumatic brain injury (TBI)

## Abstract

A hexanucleotide repeat expansion (HRE) in *C9orf72* is the most common genetic cause of amyotrophic lateral sclerosis (ALS) and frontotemporal dementia (FTD). However, patients with the HRE exhibit a wide disparity in clinical presentation and age of symptom onset suggesting an interplay between genetic background and environmental stressors. Neurotrauma as a result of traumatic brain or spinal cord injury has been shown to increase the risk of ALS/FTD in epidemiological studies. Here, we combine patient-specific induced pluripotent stem cells (iPSCs) with a custom-built device to deliver biofidelic stretch trauma to *C9orf72* patient and isogenic control motor neurons (MNs) *in vitro*. We find that mutant but not control MNs exhibit selective degeneration after a single incident of severe trauma, which can be partially rescued by pretreatment with a *C9orf72* antisense oligonucleotide. A single incident of mild trauma does not cause degeneration but leads to cytoplasmic accumulation of TDP-43 in *C9orf72* MNs. This mislocalization, which only occurs briefly in isogenic controls, is eventually restored in *C9orf72* MNs after 6 days. Lastly, repeated mild trauma ablates the ability of patient MNs to recover. These findings highlight alterations in TDP-43 dynamics in *C9orf72* ALS/FTD patient MNs following traumatic injury and demonstrate that neurotrauma compounds neuropathology in *C9orf72* ALS/FTD. More broadly, our work establishes an *in vitro* platform that can be used to interrogate the mechanistic interactions between ALS/FTD and neurotrauma.

## INTRODUCTION

Amyotrophic lateral sclerosis (ALS) is a progressive neurodegenerative disease with no effective therapeutic treatments [1]. It selectively affects upper and lower motor neurons (MNs), with cell death attributed to the disruption of an array of cellular processes including RNA metabolism, protein degradation pathways, cytoskeletal homeostasis, and neuronal excitability [2, 3]. Approximately 10% of ALS cases are caused by inherited genetic mutations, designated as familial ALS (fALS), while the remaining cases are classified as sporadic ALS (sALS) and likely arise from an interaction between genetic predisposition and environmental factors [4–6]. Roughly 30–50% of ALS patients also suffer from frontotemporal dementia (FTD), which is characterized by neurodegeneration in the frontal and temporal cortex and associated with speech and executive dysfunction [1]. The most prevalent genetic cause of ALS and FTD is an intronic hexanucleotide repeat expansion (HRE; GGGGCC_n_) in *C9orf72*, which is present in 40–50% of fALS and 5–10% of sALS cases, as well as up to 50% of all FTD cases [7–10]. The *C9orf72* HRE causes a reduction in C9orf72 protein levels and propagates deleterious gain-of-function effects, driven by the formation of C9-HRE RNA foci and the aggregation of dipeptide repeat (DPR) proteins that are transcribed and translated from the HRE likely through noncanonical mechanisms [11–17].

Patients carrying the *C9orf72* HRE are characterized by a diverse clinical presentation with a subset of individuals exhibiting only ALS or only FTD symptoms, while others are diagnosed with both [7–10]. The age of onset for C9orf72 patients is also highly variable and can range between 40 to 90 years, while in rare cases C9-HRE carriers never exhibit disease symptoms [18–23]. While the length of the C9-HRE can range between tens to thousands of repeats, the age of onset does not definitively correlate with any specific C9-HRE associated pathology such as repeat length, prevalence of RNA foci or DPR aggregates [15, 24–27]. This pronounced heterogeneity suggests that genetic interactions and environmental determinants play a significant role in shaping disease prognosis and pathogenesis in C9-HRE ALS/FTD patients.

Traumatic brain injury (TBI) caused by a blow to the head or body has been repeatedly highlighted in epidemiological studies as a significant environmental risk factor for developing neurodegenerative diseases like ALS and FTD [28–30]. TBI is among the most prevalent neurological diagnoses, impacting as many as 3 million Americans annually [31, 32]. The clinical spectrum of TBI spans a wide continuum of severity that can include patients with enduring and chronic symptoms [33, 34]. Even instances of seemingly mild traumatic events have the potential to instigate downstream pathological changes in the brain and spinal cord. This has been demonstrated prominently in cases of chronic traumatic encephalopathy (CTE), a complex and progressive neurodegenerative disease caused by repetitive but usually mild brain injuries [35, 36]. The association between TBI and ALS/FTD is further exemplified by the shared neuropathology of TAR DNA-binding protein 43 (TDP-43) aggregation [37–40]. TDP-43 is a predominantly nuclear RNA binding protein involved in RNA transport and splicing, that becomes depleted from the nucleus and forms cytoplasmic aggregations in the overwhelming majority of ALS (~97%) and as many as 50% of all FTD cases [41–43]. Notably, widespread TDP-43 aggregation is also seen in the brain and spinal cord of patients who have suffered TBI and CTE [44–46]. While these observations indicate a potential link between these diverse neurodegenerative diseases, it is unclear whether the mechanisms that drive TDP-43 pathology in ALS and TBI are shared or divergent. What also remains unclear is whether neurotrauma resulting from traumatic brain or spinal cord injury can trigger ALS disease pathophysiology and conversely, whether an ALS genetic background confers heightened vulnerability to neurotrauma, specifically in the context of human neurons.

To address these critical gaps in knowledge we established an *in vitro* model system of *C9orf72* ALS patient-specific induced pluripotent stem cell (iPSC) derived motor neurons (MNs), which can be physically traumatized by applying highly controlled and biofidelic stretch-induced injury [47–49]. We find that the *C9orf72* HRE renders patient-derived MNs more susceptible to mild and severe traumatic injury reflected in selective degeneration and altered TDP-43 localization dynamics. These pathologies were conspicuously absent from isogenic control MNs and could be partially rescued by preemptive administration of an antisense oligonucleotide (ASO) targeting the C9-HRE. We also find that repeated mild trauma ablates the ability of C9-HRE patient MNs to effectively recover and causes TDP-43 pathology and degeneration. These findings highlight alterations in TDP-43 dynamics in *C9orf72* ALS/FTD patient MNs following traumatic injury and establish an *in vitro* platform that can be used to interrogate the mechanistic interactions between ALS/FTD and neurotrauma.

## RESULTS

### Establishing an *in vitro* model of neurotrauma

**Figure 1.**
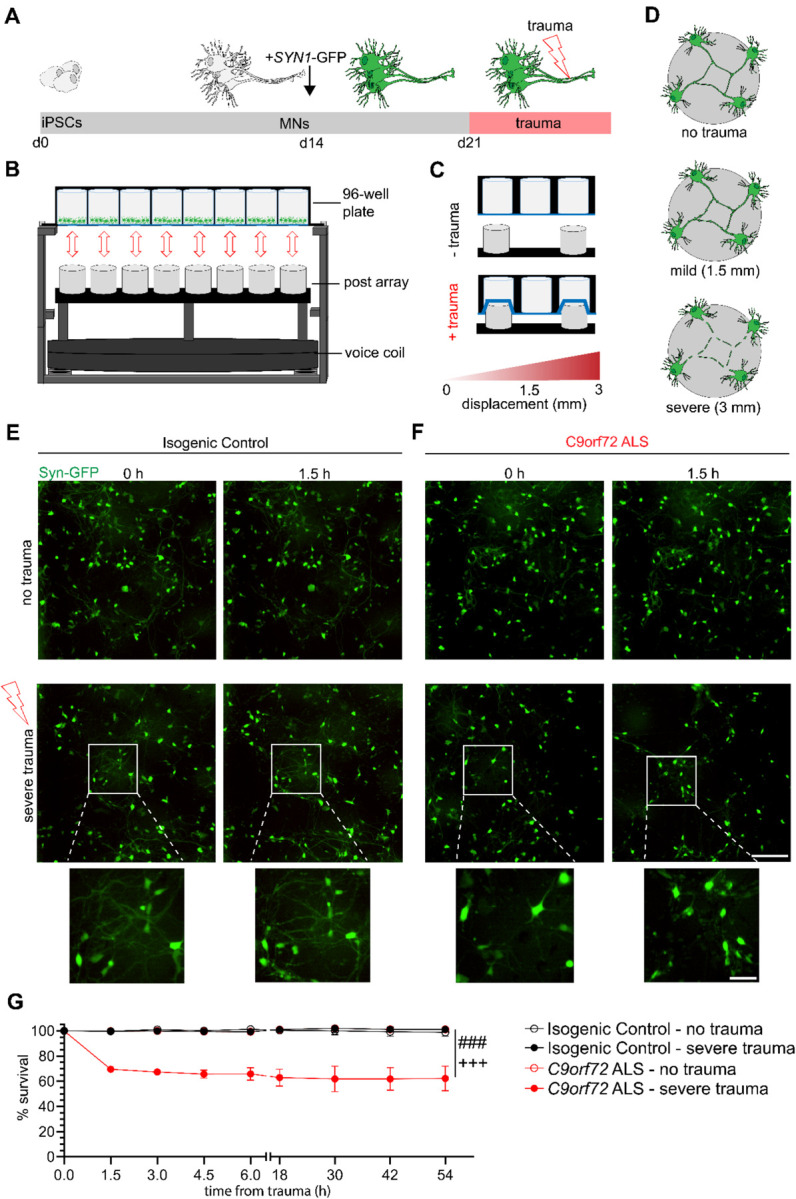
*C9orf72* ALS motor neurons show selective degeneration following severe stretch trauma. **(A)** Differentiation schematic. Motor neurons are generated from iPSCs across 14 days, then transduced with *SYN1*-GFP. One week after transduction, trauma was performed. **(B)** Experimental apparatus for induction of stretch trauma. A voice coil drives the post array into a PDMS-bottom plate. **(C)** Visual schematic of stretch trauma. PDMS stretches as posts push up before returning to original conformation. **(D)** Visual schematic of varying degrees of trauma. **(E-F)** Live imaging of *SYN1*-GFP motor neurons immediately prior (0 h) and 1.5 hours after either no trauma (NTC) or severe trauma (3 mm) for either isogenic control (**E**) or *C9orf72* (**F**) motor neurons. Cutout shows neuralization after severe trauma. Scale bar = 200 μm; inset scale bar = 50 μm **(G)** Quantification of percent survival of neurons within the frame of imaging across intervals of 1.5 hours for the first 6 hours and every 12 hours thereafter for a total of 54 hours. Experimental averages of 3 independent differentiations with 8 total fields of view. *** = p < .0005 for two-way ANOVA. Tukey’s multiple comparison tests for *C9orf72* ALS – 3mm: p < .005 compared to Isogenic Control – no trauma (###) and Isogenic Control – 3mm (+++).

The physical injury of the brain or the spinal cord can cause primary localized defects including neurodegeneration, axonal severing and edema followed by a range of downstream pathological conditions such as excitotoxicity and inflammation [31, 32]. Diffuse axonal injury (DAI) that ranges between stretching and shearing is caused by the impact of the mechanical force and the vigorous shaking of the brain and the spinal cord within the central nervous system [50–54]. To simulate neurotrauma and specifically DAI *in vitro*, we used a recently developed instrument that can deliver biofidelic stretch injury to cultured neurons [49, 55]. We selected a well-characterized set of a *C9orf72* ALS/FTD patient iPSC line and its corresponding isogenic control (Table 1 and Methods), generated by CRISPR/Cas9 gene editing, and differentiated them into ISL1/2/MAP2 positive MNs using established protocols (Fig. 1A, Supplementary Fig. 1) [56]. Upon differentiation, MNs were plated into 96-well plates with a flexible polydimethylsiloxane (PDMS) bottom and a feeder layer of primary mouse glial cells. To traumatize the cultures of MNs, plates were positioned above a post array that allowed high-speed, regulated trauma at controlled displacements of individual wells comparable to clinically relevant values (Fig. 1B–C) [49, 55]. Upward drive of the post array caused extension of the PDMS membrane in both the horizontal and vertical orientations (Supplemental Video S1). Critically, stretch trauma of one well does not affect adjacent wells, allowing direct side-by-side comparisons of conditions (Supplemental Video S2), while the instrument can inflict injury of varying ranges. In accordance with previously characterized Lagrangian strain values, displacements of 1.5 mm and 3 mm were classified as mild or severe, respectively (Fig. 1D) [49, 55, 57].

### *C9orf72* ALS MNs selectively degenerate following severe stretch trauma

To specifically visualize iPSC-derived MNs using live imaging analysis, we transduced the cultures with a lentivirus reporting GFP under the Synapsin 1 promoter (*SYN1*-GFP) following their plating on PDMS and prior to injury (Fig. 1A). We first asked whether a single incident of severe trauma (3 mm) would inflict differential responses between patient and isogenic control MNs. Quantification of *SYN1*-GFP cells revealed that *C9orf72* ALS MNs displayed markedly reduced viability of 70% at 1.5 hours post-trauma relative to the no trauma control condition, followed by a persistent gradual decline reaching 61% approximately 2 days later (no trauma control *Vs*. severe trauma in *C9orf72*; N=3, p< 0.0001) (Fig. 1E–G). In stark contrast, the isogenic control MNs injured at the same time exhibited no significant changes in viability even after 2 days (no trauma control *Vs*. severe trauma in controls; N=3, p=NS) (Fig. 1E–G). These data demonstrate that the *C9orf72* ALS MNs selectively degenerate following severe stretch trauma.

**Figure 2.**
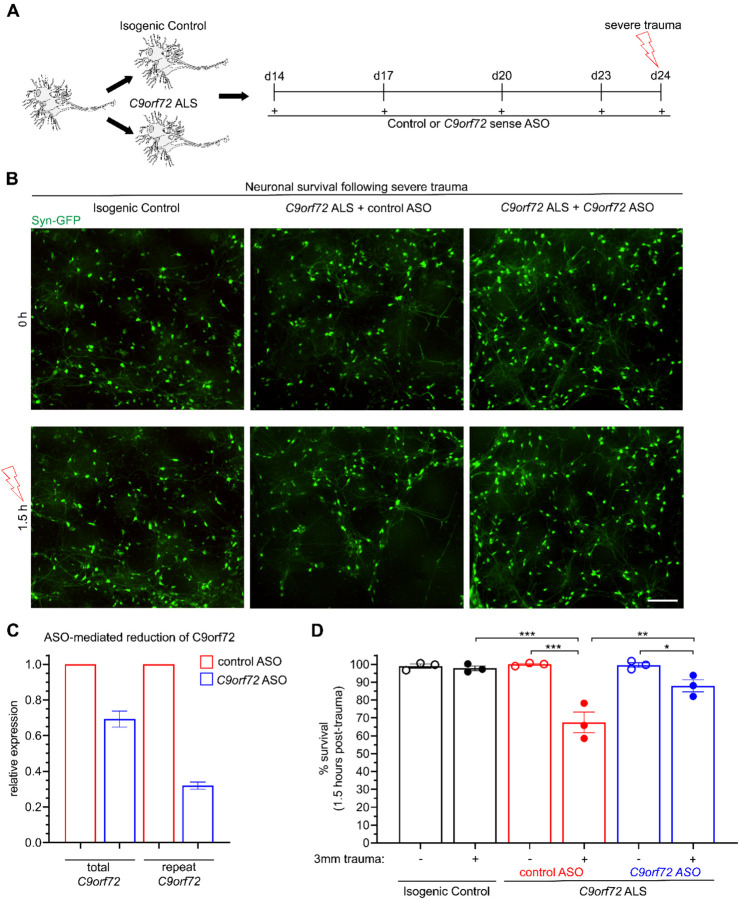
Neurodegeneration following severe trauma is partially rescued by pretreatment with an ASO targeting the *C9orf72* repeat expansion. **(A)** Experimental schematic for ASO treatment prior to severe trauma. **(B)** Live imaging of *SYN1*-GFP MNs 1.5 hours following severe trauma or no trauma. Scale bar = 200 μm. **(C)** Relative expression of total *C9orf72* and repeat-associated *C9orf72* in *C9orf72* ALS MNs following treatment from a *C9orf72* ASO specifically targeting the repeat expansion compared to a scramble ASO from 2 biological replicates (1 differentiation). **(D)** Quantification of percent survival 1.5 hours following trauma, showing differences between *C9orf72* ALS pretreated with a scramble ASO (red) and a repeat-targeting ASO (blue). Experimental averages of 3 independent differentiations from 16 fields of view. Two-way ANOVA p < .0005. For Tukey’s multiple comparisons test, * = p < .05 ** = p < .01 *** = p < .005.

To ascertain whether the heightened susceptibility to severe trauma was intrinsically linked to the *C9orf72* HRE, we subjected the MN cultures to a pre-treatment with either a scrambled or an ASO targeting the sense *C9orf72* repeat expansion and repeated the experimental paradigm of severe injury (Fig. 2A–B). Consistent with previous findings [58], we found that the ASO reduced the expression of both the sense repeat and total *C9orf72* transcript by approximately 70% and 30% respectively, relative to samples treated with a scrambled, control ASO (Fig. 2C). Monitoring of *SYN1*-GFP neurons 1.5 hours after trauma revealed a 30% reduction in the *C9orf72* cultures, whereas as previously observed the isogenic controls were unaffected (control trauma *Vs*. C9 trauma; N=3, p< 0.0001) (Fig. 2B, 2D). Notably, pretreating the *C9orf72* ALS MNs with the HRE-targeting ASO caused a significant but incomplete rescue of neurodegeneration following severe trauma, suggesting that the sense repeat RNA is in part responsible for MN vulnerability to neurotrauma (scrambled ASO trauma *Vs*. C9 ASO trauma in mutant *C9orf72* MNs; N=3, p< 0.005) (Fig. 2B, 2D).

### Mild stretch trauma increases the abundance of G_4_C_2_ RNA foci but not GP-DRP in *C9orf72* ALS MNs

**Figure 3.**
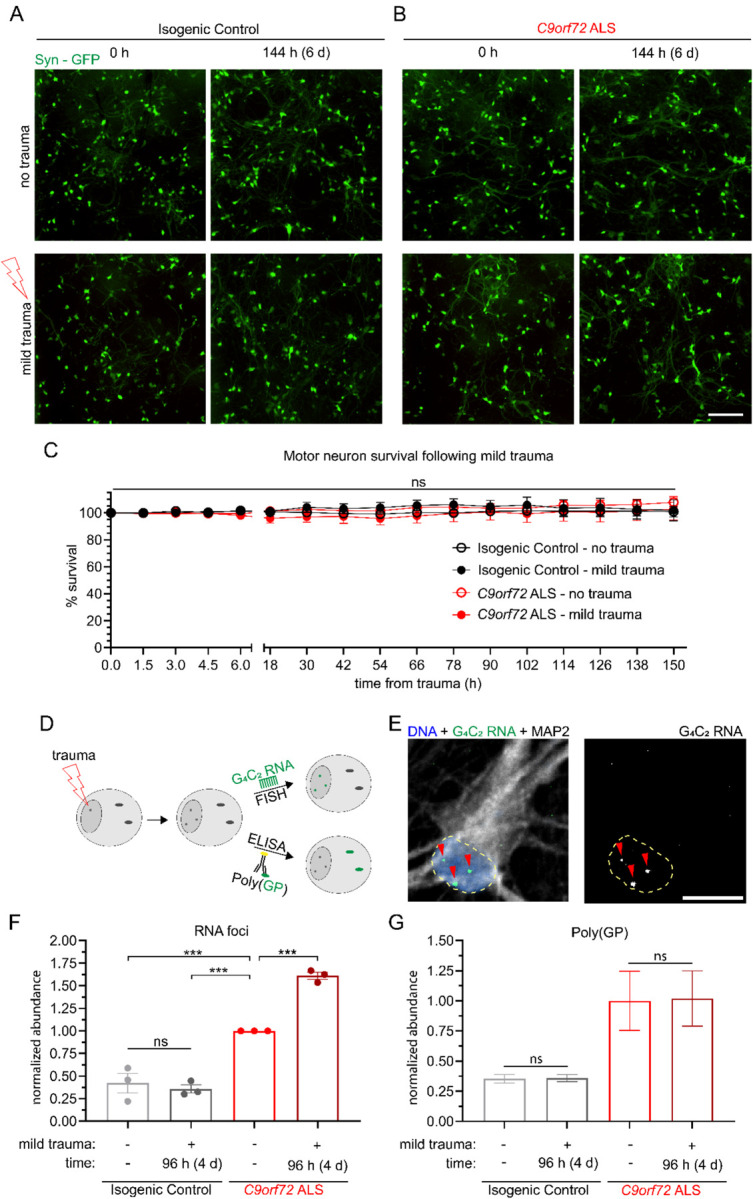
Mild trauma increases incidence of RNA foci but not DPR production in *C9orf72* ALS motor neurons. **(A-B)** Live imaging of synapsin-GFP isogenic control motor neurons across a period beyond 6 days (150 hours) following either no trauma or mild trauma (1.5 mm) in either isogenic control (**A**) or *C9orf72* ALS (**B**) motor neurons. Scale bar = 200 μm. **(C)** Quantification of percent survival for over 6 days (150h) following mild trauma. Two-way ANOVA NS. **(D)** Experimental schematic for quantification of toxic gain of function *C9orf72* pathologies. **(E)** Representative image of FISH probe analyzed for RNA foci. **(F)** Quantification of percent of neurons with RNA foci 4 days following mild trauma relative to *C9orf72* ALS NTC. Experimental averages for 3 independent differentiations. Two-way ANOVA p < 0.0005. Tukey’s multiple comparisons test *** = p < 0.005 relative to *C9orf72* ALS NTC. **(G)** ELISA quantification of poly(GP) DPR 4 days following mild trauma.

Having established an increased susceptibility of *C9orf72* MNs to severe trauma, we next sought to characterize pathological changes under conditions of trauma that did not elicit immediate degeneration. We first examined *C9orf72* and isogenic control MN cultures over an extended time course of over 144 hours (6 days) after a single incidence of mild traumatic injury (1.5 mm). This analysis showed that neither mutant nor control MNs were affected, indicating the absence of a delayed or secondary wave of degeneration (no trauma *Vs*. mild trauma; N=3, p=NS) (Fig. 3A–C). We next interrogated the effects of mild sublethal injury to *C9orf72*-associated gain-of-function neuropathology by conducting FISH and ELISA assays targeting C9-HRE RNA foci and poly-GP DPR production, respectively (Fig. 3D). As expected, the abundance of nuclear (G_4_C_2_)n RNA foci was found to be significantly higher in C9-HRE patient MNs at baseline. Mild trauma induced a 50% increase in the number of C9-HRE patient MNs with G_4_C_2_ RNA foci after 4 days, while the controls were unaffected (no trauma *Vs*. mild trauma in *C9orf72*; N=3, p< 0.0001) (Fig. 3E–F and Supplementary Fig. 2). Intriguingly, this effect was not accompanied by a corresponding increase in the relative amount of poly-GP, suggesting that trauma does not induce non-ATG dependent translation unlike other forms of cellular stress (no trauma *Vs*. mild trauma in *C9orf72*; N=3, p=NS) (Fig. 3G) [59].

### Stretch trauma induces alterations in the nucleocytoplasmic localization of TDP-43

**Figure 4.**
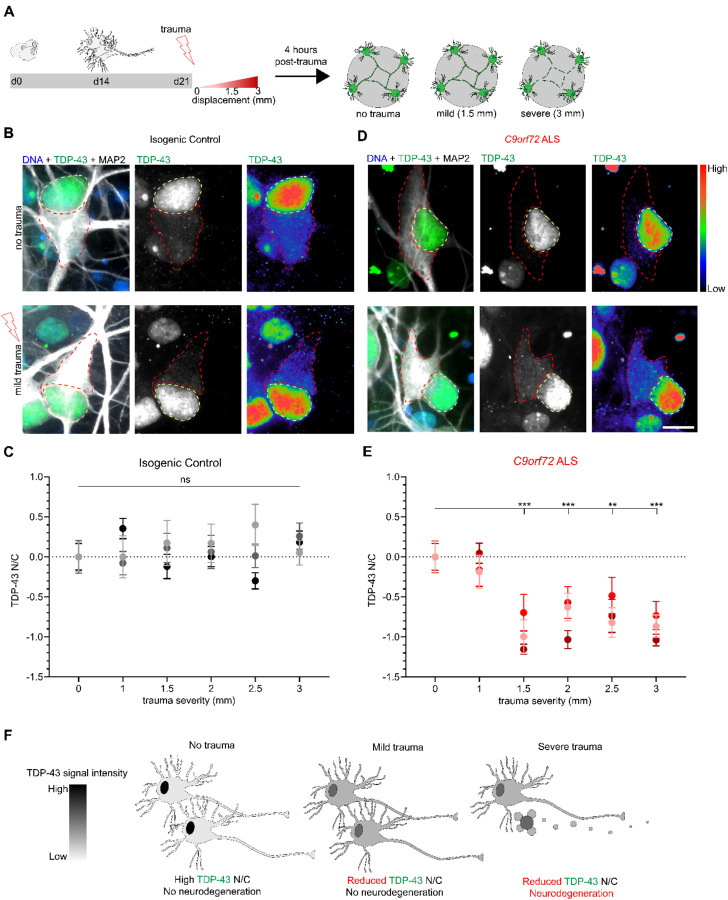
Traumatic injury induces TDP-43 mislocalization across a range of severities in *C9orf72* ALS but not isogenic control motor neurons. **(A)** Schematic depicting paradigm for stretch trauma across a range of severities. **(B and D)** Immunocytochemistry shows TDP-43 distribution in either isogenic control (**B**) or *C9orf72* ALS (**D**) motor neurons prior to and 4 hours post-trauma. No trauma compared to 1.5mm displacement. Scale bar = 10 μm. **(C and E)** Z-score quantification of TDP-43 N/C at each displacement value relative to no trauma in either isogenic control (**C**) or *C9orf72* ALS (**E**) motor neurons. Data represent experimental averages from 3 independent differentiations for a total of 84 – 91 (**C**) or 58 – 87 (**E**) cells. (**C**) One-way ANOVA NS (**E**) One-way ANOVA p < 0.0005. Tukey’s multiple comparisons test *** p < 0.005, ** p < 0.01. **(F)** Schematic representation of mild and severe trauma compared to no trauma with respect to TDP-43 N/C and viability.

The cytoplasmic mis-localization of TDP-43 constitutes an essential convergent pathology between ALS and neurotrauma resulting from TBI, as observed in CTE cases [60, 61]. To assess the dynamics of TDP-43 in the context of the *in vitro* stretch injury model, we next subjected C9-HRE patient and isogenic control MNs to trauma spanning a spectrum of severities (0, 1, 1.5, 2, 2.5, and 3 mm membrane displacement) and quantified the nucleocytoplasmic (N/C) localization of TDP-43 by immunocytochemistry and confocal imaging 4 hours post-trauma (Fig. 4A). The control MNs did not exhibit any significant alteration in the N/C ratio of TDP-43 across any of the trauma severities we examined (no trauma *Vs*. trauma in controls; N=3, M=84–91 cells, p=NS) (Fig. 4B–C and Supplementary Fig. 3A–B). In stark contrast, we found a significant reduction in the N/C ratio of TDP-43 in response to trauma equal or stronger to 1.5 mm in C9-HRE patient-derived MAP2 positive MNs (no trauma *Vs*. trauma in *C9orf72*; N=3, M=58–88 cells, p<0.005) (Fig. 4D–E and Supplementary Fig. 3A and C). Notably, this shift in N/C TDP-43 signal was not dose-dependent but rather threshold dependent. To further assess the specificity of this effect, we interrogated mutant *SOD1*^*A4V/+*^ and isogenic control MN cultures derived from a well characterized set of ALS patient iPSC lines [62–64] that were subjected to the same wide range of traumatic injury paradigm (Supplementary Fig. 4). Crucially, the *SOD1*^*A4V/+*^ MNs did not display any discernable reduction in the N/C TDP-43 ratio in these experiments (no trauma *Vs*. trauma in *SOD1*^*A4V/+*^; N=3, M=91–101 cells, p=NS), in accordance with the fact that *SOD1* ALS patients fall within the 3% of all patients that do not develop neuropathological TDP-43 aggregates postmortem [65, 66].

**Figure 5.**
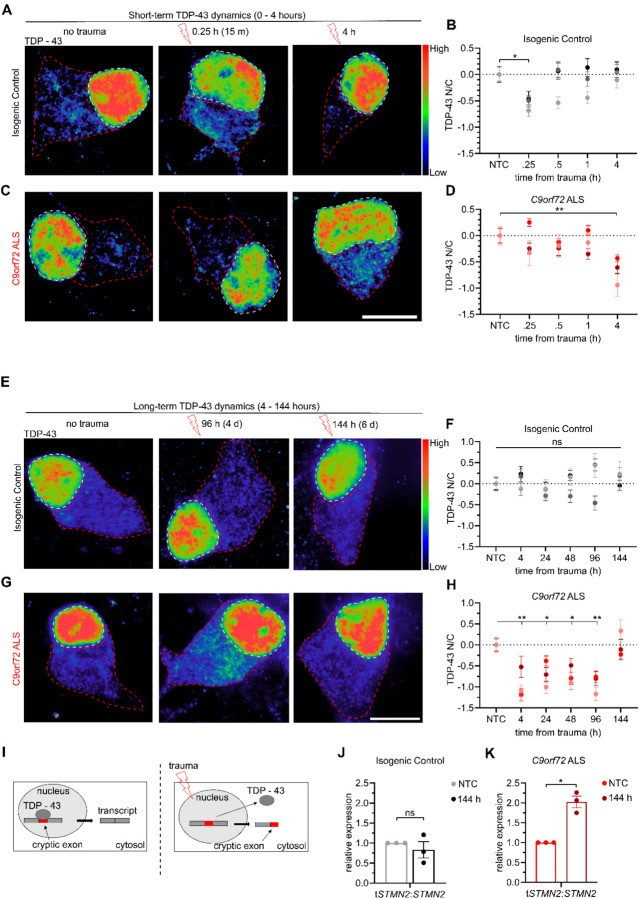
Mild trauma induces extended TDP-43 mislocalization and increased mis-splicing of STMN2 in *C9orf72* ALS motor neurons. **(A and C)** Immunocytochemistry showing TDP-43 in either isogenic control (**A**) or *C9orf72* ALS (**C**) motor neurons at 0 hours, 15 minutes (0.25 h), and 4 hours following mild trauma. Scale bar = 10 μm **(B and D)** Z-score quantification of TDP-43 N/C relative to no trauma in either isogenic control (**B**) or *C9orf72* ALS (**D**) motor neurons. Data represent experimental averages from 3 independent differentiations for a total of 133 – 162 (**B**) or 132 – 139 (**D**) cells. **(B)** One-way ANOVA p < 0.05. Tukey’s multiple comparisons test * = p < 0.05. (**D**) One-way ANOVA p < 0.05. Tukey’s multiple comparisons test ** = p < 0.01. **(E and G)** Immunocytochemistry showing TDP-43 in either isogenic control (**E**) or *C9orf72* ALS (**G**) motor neurons at 0 hours, 96 hours, and 144 hours following mild trauma. Scale bar = 10 μm **(F and H)** Z-score quantification of TDP-43 N/C relative to NTC in either isogenic control (**F**) or *C9orf72* ALS (**H**) motor neurons. Data represent experimental averages from 3 independent differentiations for a total of 118 – 133 (**F**) or 123 – 138 (**H**) cells. (**F**) One-way ANOVA NS. (**H**) One-way ANOVA p < .0005. Tukey’s multiple comparisons test * = p < 0.05, ** = p < 0.01. **(I)** Visual schematic of mis-splicing caused by mild trauma. **(J and K)** Quantification of relative expression of truncated *STMN2* (t*STMN2*) relative to total expression (*tSTMN2/STMN2*) by qPCR for Isogenic Control (**J**) or *C9orf72* ALS (**K**). Data for each line are normalized relative to no trauma. Data represent 3 independent differentiations analyzed by Welch’s t-test. * = p < 0.05.

Our findings demonstrate that TDP-43 exhibits a significant shift in N/C localization 4 hours after severe or even mild traumatic injury without any measurable effects on neuronal viability, but only in the context of a relevant ALS genetic background (Fig. 4F). To better define the dynamics of this mis-localization we quantified the N/C ratio of TDP-43 in C9-HRE patient and isogenic control MNs with higher temporal resolution after a single mild traumatic insult. We found that isogenic control MNs exhibited a brief reduction in TDP-43 N/C ratio 15 minutes post-trauma that normalized to baseline levels by 30 minutes (no trauma *Vs*. trauma in controls at 15 min; N=3, M=177 cells, p<0.05) (Fig. 5A–B). In contrast, this post-traumatic shift in TDP-43 was delayed in *C9orf72* ALS MNs and did not occur until 4 hours (no trauma *Vs*. trauma in *C9orf72* at 4 hrs; N=3, M=170 cells, p<0.01) (Fig. 5C–D) as we previously described (Fig. 4D). The observation of a brief shift in TDP-43 localization followed by a full recovery in control MNs prompted us to next ask whether a similar recovery occurs at all in mutant *C9orf72* ALS MNs. Thus, we repeated the mild traumatic injury and carefully quantified the localization of TDP-43 for up to 6 days post injury. The control MNs exhibited a consistent N/C ratio in TDP-43 throughout this extended time course (Fig. 5E–F). The *C9orf72* ALS MNs showed a sustained level of TDP-43 misloclaization, that peaked at 4 hours and recovered to pre-traumatic levels only after 6 days (no trauma *Vs*. trauma in *C9orf72* at 4 hrs p<0.01; at 96 hours p<0.01; at 144 hours p=NS; N=3, M=127,133, and 124 cells respectively) (Fig. 5G–H and Supplementary Fig. 5). Taken together, these findings underscore the potential for TDP-43 mislocalization to be a normative response to traumatic injury. However, the significantly delayed dynamics of the response and ultimate delayed recovery in C9-HRE MNs indicates that the ALS genetic background compromise the response to neurotrauma.

Dysfunction of TDP-43 has recently been shown to increase the mis-splicing of a series of human-specific target mRNAs that appear to be regulated post-transcriptionally such as *STMN2*, which encodes a regulator of microtubule homeostasis, and *UNC13A*, which is involved in synaptic vesicle release [67–71]. Binding of TDP-43 to these mRNAs prevents mRNA decay and downregulation (Fig. 5I) To determine whether the prolonged mis-localization of TDP-43 induced by mild traumatic injury recapitulated aberrant RNA splicing, we employed qRT-PCR assays to quantify the level of cryptic exon inclusion in *STMN2* [71, 72]. We found that in the isogenic control MN cultures the relative proportion of truncated *STMN2* (*tSTMN2*) relative to full-length *STMN2* was unchanged 144 hours after injury (Fig. 5J). In contrast, *tSTMN2/STMN2* was moderately but significantly increased 144 hours following trauma in *C9orf72* ALS MNs (Fig. 5K). Notably, this occurs even though the N/C distribution of TDP-43 has returned to baseline levels at this point, suggesting that neurotrauma can cause lingering defects in mRNA splicing even beyond recovery of nuclear localization of TDP-43.

### Repetitive mild stretch trauma causes neurodegeneration and prevents recovery of TDP-43 mislocalization in *C9orf72* ALS MNs

**Figure 6.**
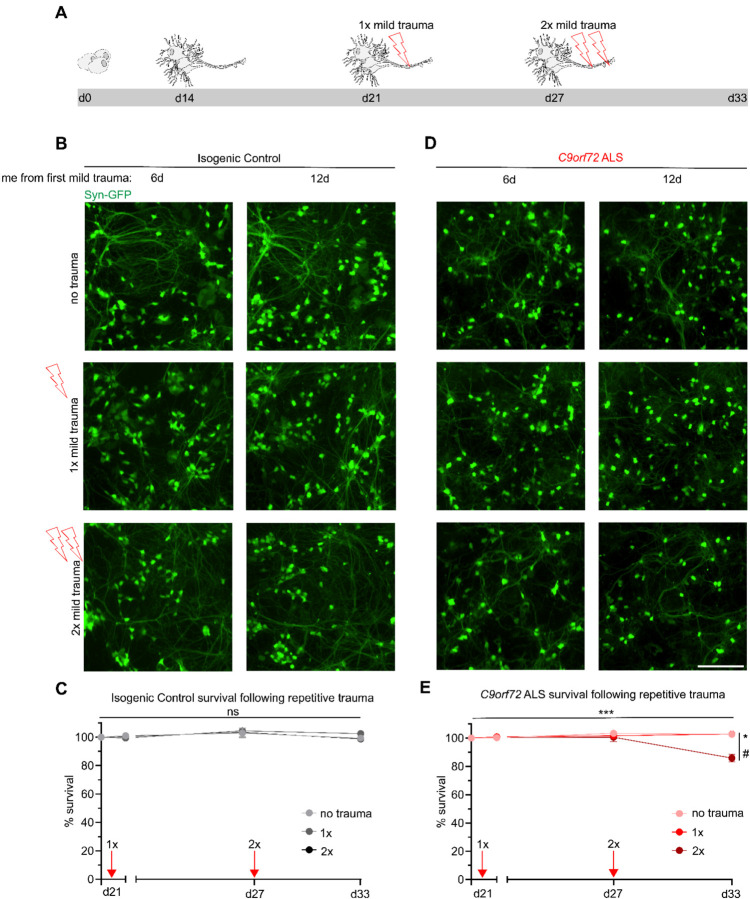
Repetitive mild trauma facilitates neurodegeneration in *C9orf72* ALS motor neurons. **(A)** Schematic depicting trauma paradigm for repetitive injury. **(B and D)** Live imaging of synapsin-GFP motor neurons following either no trauma, 1x mild trauma or 2x mild trauma across an extended period of time (12 days from 1^st^ trauma) in isogenic control (**B**) or *C9orf72* ALS (**D**). Scale bar = 200 μm. **(C and E)** Quantification of synapsin-GFP motor neurons in isogenic control (**C**) or *C9orf72* ALS (**E**) following trauma. Induction of mild trauma is indicated as a red arrow on the graph. Data represent 9 fields of view from 3 independent differentiations. (**E)** Two-way ANOVA; p < 0.0005. Tukey’s multiple comparisons test 2x vs NTC; * p <0.05 and 2x vs 1x # p < 0.05.

**Figure 7.**
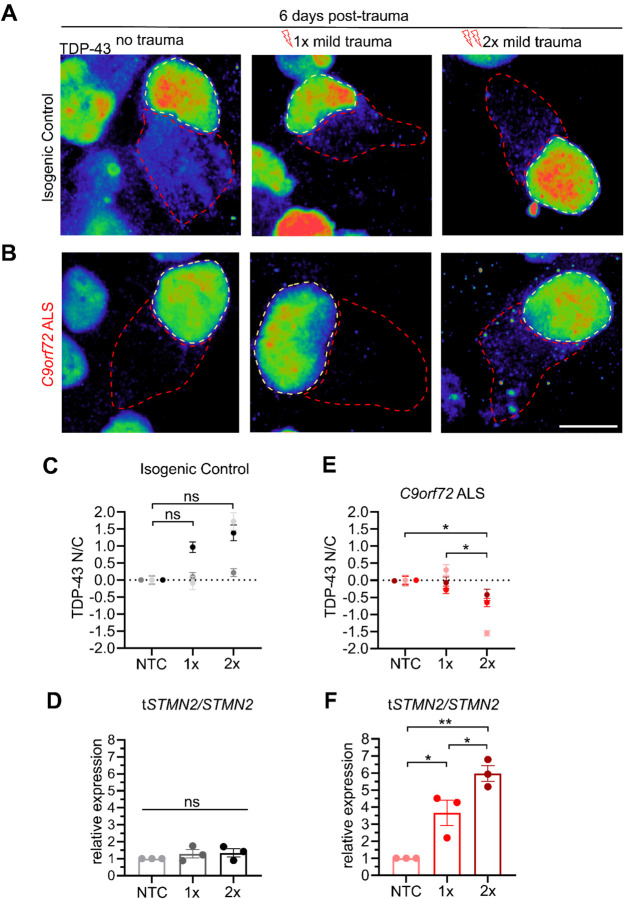
Repetitive mild trauma prevents TDP-43 recovery in *C9orf72* ALS motor neurons. **(A-B)** TDP-43 staining in cells 6 days post-trauma following either no trauma, 1x mild trauma, or 2x mild trauma in isogenic control (**A**) or *C9orf72* ALS (**B**) motor neurons. Scale bar = 10 μm. **(C and E)** Quantification of TDP-43 N:C 6 days following mild traumas for either isogenic control (**C**) or *C9orf72* ALS (**E**) motor neurons. (C) One-way ANOVA; NS. (E) One-way ANOVA; p < 0.05. Tukey’s multiple comparisons test relative to NTC indicated with * = p < 0.05; *** = p < 0.0005. 169–184 neurons from 3 independent differentiations. **(D and F)** Quantification of relative expression of truncated STMN2 (tSTMN2) relative to STMN2 6 days post-trauma following either no trauma, 1x mild trauma, or 2x mild trauma for isogenic control (**D**) or *C9orf72* ALS (**F**) motor neurons. 3 biological replicates per conditions. (D) One-way ANOVA; NS. (F) One-way ANOVA; p < 0.005. Tukey’s multiple comparisons indicated * = p < 0.05 and ** = p < 0.01.

The resilience of control MNs to stretch induced injury as well as the ability of *C9orf72* ALS MNs to ultimately restore TDP-43 localization following mild injury prompted us to next investigate their susceptibility and molecular responses to repeated incidences of trauma. We designed an experimental paradigm where MNs were injured mildly on day 21, analyzed and allowed to recover for 6 days, injured again using the same mild force (1.5 mm), followed by another 6 days of recovery and analysis (Fig. 6A). MNs were afforded a 6-day recovery period to match the duration for TDP-43 recovery following acute trauma, while downstream pathologies like C9-HRE RNA foci and aberrant mRNA splicing may persist (Figs. 3F and 5K). Intriguingly, introducing a second instance of mild trauma proved sufficient to significantly reduce survival of *C9orf72* ALS MNs by approximately 10% (no trauma control *Vs*. 2x mild trauma in C9orf72; N=3, p<0.05), while isogenic control MNs continued to be resilient, as their numbers remained unaffected (no trauma control *Vs*. 2x mild trauma in controls; N=3, p=NS) (Fig. 6B–E). Quantification of the N/C localization of TDP-43 in control MNs showed no effect after a single or a second incidence of mild trauma (no trauma control *Vs*. 1x or 2x mild trauma in controls; N=3, M=204 and 211 cells, p=NS) (Fig. 7A, C and Supplementary Fig. 6). In accordance with this observation, there was no shift in the *tSTNM2/STMN2* transcript ratio (Fig. 7E).

At the same time, in *C9orf72* ALS MNs the N/C distribution of TDP-43 was restored 6 days after one mild hit as we observed previously (Fig. 5G–H). However, a subsequent mild hit compromised their ability to effectively recover and caused substantial reduction in N/C TDP-43 signal (no trauma control *Vs*. 1x mild trauma in *C9orf72* p=NS; Vs. 2x p<0.05; N=3, M=196 cells) (Fig. 7B, D and Supplementary Fig. 6). Critically, this defect was reflected in the abundance of *tSTMN2*, with a significantly increased accumulation after a second mild traumatic injury relative to a single acute mild trauma (Fig. 7F). Collectively, these data highlight the compounding effect of multiple injuries of a mild nature on neurodegenerative molecular pathologies and susceptibility of mutant *C9orf72* MNs.

## DISCUSSION

A series of comprehensive epidemiological studies have documented the increased prevalence of ALS in populations exposed to various environmental stressors including traumatic injury [73–76]. However, all epidemiological studies are correlational by nature, preventing an explicit link from being drawn. Within this premise, we sought to develop a human iPSC-based preclinical model that would allow us to functionally interrogate the interaction between neurotrauma, degeneration, and associated molecular pathology in the context of patient human ALS MNs. We selected to use MNs with a *C9orf72* HRE on account of the wide range of clinical diversity associated with these patients [18–23], and implemented a custom built mechanical instrument that can deliver biofidelic stretch-based traumatic injury *in vitro* [47–49]. We found that our model faithfully recapitulated core tenets of *C9orf72* ALS pathology in both a transient and persistent manner. *C9orf72* ALS patient MNs were selectively vulnerable to trauma unveiling a distinct susceptibility profile that underscores the complex interplay between genetic predisposition and environmental influences in shaping disease manifestation.

The mechanistic association between ALS and TBI has been investigated using animals including *Drosophila* [61, 77] and rodent models of disease [78–82]. In some of these instances, TBI has been shown to cause stress granule formation, altered N/C transport dynamics, and TDP-43 mis-localization independent of genetic background [46, 60, 61, 78, 82], pathologies that have been documented in ALS patients [38, 40, 41, 83–88]. Moreover, while there are other reports of utilizing iPSC technologies to model TBI mechanisms *in vitro* [89–92] our approach represents the first to combine ALS patient specific iPSC-derived neurons with a traumatic injury infliction. The HRE in *C9orf72*, which represents the largest genetic cause of ALS and FTD leads to dysfunction and eventual neurodegeneration through both loss-of-function effects as well as gain-of-function RNA and DPR associated toxicity [11–17]. Our findings suggest that the increased vulnerability of *C9orf72* neurons to TBI is primarily mediated by HRE RNA associated toxicity. This is supported by the fact that mild trauma elicited an increase in RNA foci but not poly-GP DPR production even 4 days after injury. This is surprising given that non-canonical translation has been shown to be triggered by the induction of the integrated stress response (ISR) and neuronal hyper-excitability [59], cellular pathways that likely occur downstream of TBI. It is possible that an increase in DPR production would be observed beyond the temporal scope of our analysis or that there is selective translation of non-GP DPRs.

We showed that targeting the C9-HRE with an ASO designed against the sense transcript was partially neuroprotective, implicating the gain-of-function effects of the mutation in the increased susceptibility to severe trauma. The incomplete rescue of the ASO could be related to the contribution of anti-sense *C9orf72* transcript driven toxicity [93, 94] or simply insufficient dosing. Nevertheless, these experiments represent one of the first examples of a pharmacological pre-treatment that can successfully prevent post-traumatic neurodegeneration in human ALS patient neurons.

Our observation that mild trauma led to extended mis-localization of TDP-43 in *C9orf72* ALS MNs, while only a brief mis-localization in control MNs has interesting implications. It suggests that the re-distribution of TDP-43 may be a physiological response to trauma and stress and is in accordance with a series of prior studies. TDP-43 has been shown to be associated with cytosolic stress granules following induced stress through chemical treatment [60, 95–97]. Furthermore TDP-43 mis-localization has been observed in animal models of axonal injury, both in cases of TDP-43 ALS-associated mutations and a wild-type genetic background [61, 78, 98, 99]. We propose that the *C9orf72* mutation compromises the typical signaling cascade downstream of injury including the normal re-localization of TDP-43. This could be caused by broad nuclear transport defects that have been previously characterized in both models of ALS [61, 83, 84, 100] and in *Drosophila* models of TBI [61]. Furthermore, the increased mis-splicing of TDP-43 target genes such as *STMN2* even at a time when TDP-43 distribution had recovered to baseline levels, suggests a sustained pathology that extends beyond the initial phase of traumatic injury and could be mediating subsequent vulnerability. This is particularly relevant in the context of repeated mild trauma, which was sufficient to induce neurodegeneration specifically in *C9orf72* ALS MNs. It is possible that nonlethal pathologies such as mis-spliced TDP-43 target genes and C9-HRE nuclear foci accumulate in cells and can ultimately contribute to neurodegeneration.

While the model system we developed here will be a valuable tool as it allows for the investigation of molecular pathology related to traumatic injury in the context of human neurons and a patient-specific genetic background, it also has some major limitations. Firstly, we exclusively focused on the effects of traumatic injury on neurons, however traumatic injury is known to cause non neuronal responses involving oligodendrocytes, and activated astrocytes and microglia [101]. Incorporation of these cell types either directly generated from iPSCs through directed differentiation strategies [102], or in the context of 3D cortical or spinal cord multicellular organoids [103, 104] would allow for a more complete investigation of traumatic injury responses. Secondly, the mechanical injury associated with TBI can come in many ways such as a physical hit, or blast, or even a penetrating injury and downstream of these forces are several tissue damaging mechanisms beyond stretch-induced injury which we modelled here including edema, inflammation etc. [105–108]. Lastly, iPSC-models *in vitro* lack the structural organization and functional connectivity of the intact nervous system in model organisms. At the same time, these limitations can also offer some value in representing a reductionist approach to isolate cell autonomous effects in response to specific types of insults such as stretch-induced injury in this case [109]. Taken together, our results corroborate the value of a preclinical model for traumatic injury, aptly employing stretch injury to simulate physiologically relevant environmental factors. Our work demonstrates the ability to recapitulate clinically relevant pathologies, characterizing the crosstalk between environmental factors and the pathophysiological trajectory of *C9orf72* ALS/FTD.

## MATERIALS AND METHODS

### Stem cell culture

Human induced pluripotent stem cells (hiPSC) *C9orf72* mutant and isogenic control pair (CS29) were obtained from the Cedars Sinai iPSC repository. Isogenic controls were generated by CRISPR/Cas9 gene editing targeting the regions immediately 5’ and 3’ of the repeat expansion. Stem cells were maintained on Matrigel (BD Biosciences) with mTeSR1 media (Stem Cell Technologies) and passaged using Accutase (Innovative Cell Technologies). All cell cultures were maintained at 37°C and 5% CO2. All iPSC cultures are tested regularly for mycoplasma. All lines used were mycoplasma-free. Passaged iPSC cultures were kept in Y27632 for 24 hours (DNSK international).

### Motor neuron differentiation

When hiPSC colonies were ~80% confluent, media was replaced with N2B27 medium (50% DMEM:F12, 50% Neurobasal, supplemented with NEAA, Glutamax, N2 and B27; Thermo Fisher Scientific) containing 10μM SB431542 (DNSK International), 100nM LDN-193189 (Bio-Techne), 1 μM Retinoic Acid (RA, Millipore-Sigma) and 1μM of Smoothened-Agonist (SAG, DNSK International). Media was changed daily. On day 6, culture media was switched to N2B27 medium supplemented with 1μM RA, 1μM SAG, 5μM DAPT (Bio-Techne) and 4μM SU5402 (DNSK International), fed daily until 14 total days of differentiation. On day 14, MNs were dissociated using TrypLE Express (Thermo Fisher Scientific) supplemented with DNase I (Worthington) and plated onto murine astrocytes seeded on a polydimethylsiloxane (PDMS) plate (Nunc) pre-coated with Poly-D-Lysine (BD Biosciences). MNs were cultured in Neurobasal medium supplemented with NEAA, Glutamax, N2, B27, Ascorbic acid (0.2 μg/ml; Sigma-Aldrich) BDNF, CNTF and GDNF (10ng/mL, Bio-Techne), 2% FBS (Gemini), and P/S (Thermo Fisher Scientific). For the first 24 hours, MNs were maintained in Y. Media was half-changed every 2–3 days after the first 24 hours.

### Simulation of stretch trauma

Prior to experimentation, a high-speed camera was used to establish a zero plane for PDMS plates, defined as the point where the posts for injury encountered the PDMS bottom but did not cause displacement in the membrane. Displacements and injuries were performed using custom software (Python) controlling the post array via a servo controller. Plates were clamped down using thick acrylic to hold the plates stationary during injury in a manner that would not contribute additional stress to the plates (the force from securing the plates was eventy distributed across the plate). Prior to injury, posts were lubricated using vegetable oil (Kroger) to minimize friction and adhesion between the posts and the PDMS during trauma. Trauma simulations spanned a period of ~70 msec. Displacements were selected based on previously calculated quantifications of Lagrangian strain.

### Live imaging of *SYN1*-GFP cells

For live imaging experiments, lentiviral *SYN1*-GFP (PZ196, Addgene) was added to neurons in suspension following dissociation on day 14. Neurons were plated in culture media with *SYN1*-GFP for the first 24 hours and following a full media change were cultured as previously described. For nuclear staining, NucSpot Live 650 Nuclear Stain (Biotium) was added 1:1000 to media 1 hour prior to imaging and supplemented with media changes. Propidium iodide (Sigma) was added to media 1:2000 and supplemented with media changes. Images were collected prior to trauma and every 1.5 hours for the first 6 hours following trauma. After 6 hours, images were collected every 12 hours. Images showing synapsin-GFP alone were collected from an IncuCyte S3 (Sartorius). Images showing PI and nuclear stains were collected using a Nikon BioStation (Nikon). Image Analysis was performed using Image J (U. S. National Institutes of Health, Bethesda, Maryland, USA)

### Antisense oligonucleotide treatment

Following MN dissociation, ASOs were added to day 14 motor neurons in suspension at a concentration of 5μM (Integrated DNA Technologies). Every 3 days, media was supplemented with 2.5μM ASOs. On the 10^th^ day, RNA was collected for qRT-PCR. Trauma experiments were performed following 10 days of ASO administration.

### RNA preparation and qRT-PCR

Cells were harvested by direct lysis in TRIzol Reagent (Life Technologies). RNA isolation was performed via phase separation according to manufacturers’ protocol. 1–2μg of RNA was used as input for generation of cDNA according to the SuperScript IV First-Strand cDNA Synthesis Reaction Kit (Thermo Fisher Scientific). RT-PCR was performed using PowerUp SYBR Green Master Mix (Thermo Fisher Scientific) on the CFX system (Bio-Rad). Two housekeeping genes (RPLP0/CYC1) were averaged for determination of the cycle of threshold (Ct). For ASO effectiveness, this Ct was subtracted from the average Ct for *C9orf72* to obtain the ΔCt. Relative gene expression was calculated as 2–ΔCt (ΔΔCt) and expressed relative to the scrambled ASO treatment. For splicing experiments, the average Ct for the gene of interest was subtracted from the average Ct for the spliced isoform for a determination of splice:total expression.

### Fluorescent in situ hybridization (FISH)

FISH was performed as previously detailed [110]. Briefly, cells were cultured in PDMS plates, fixed with 4% PFA, and permeabilized with 0.3% Triton for 15 minutes. Prior to hybridization, cells were equilibrated in 50% formamide (Spectrum) + 2X SSC (Invitrogen) for 10 minutes at 60°C. During equilibration, probe mixture was preheated at 95°C for 10 minutes. To hybridize, 27 μL of probe mixture in 200 μL of hybridization buffer were added to each well, and cells were incubated at 60°C for 1 hour. Following hybridization, cells were incubated with 50% formamide + 2X SSC at 65°C for 25 minutes, then with fresh 50% formamide + 2X SSC at 65°C for 15 minutes, and finally with 1X SSC + 40% formamide at 60°C for 10 minutes. Cells then underwent a series of room temperature washes: 3x with 1X SSC, 2× 5 minutes with 1X SSC, 1× 5 minutes with Tris-buffered saline (Invitrogen), and finally 1× 5 minutes with Tris-glycine buffer (Invitrogen). Cells were immersed in 3% PFA for post-fixation and then incubated for 1 hour with blocking buffer at room temperature. Cells were incubated in immunofluorescence buffer with primary antibodies at 4°C overnight. Following a brief wash in immunofluorescence buffer, cells were placed in immunofluorescence buffer with secondary antibodies for 30 minutes at room temperature. Following incubation with secondary antibodies, cells were sequentially washed with immunofluorescence buffer, Tris-buffered saline, Tris-glycine buffer, 2mM MgCl2 in PBS, and PBS. DAPI (Invitrogen) was added to wells in PBS and imaging was performed immediately after.

Hybridization buffer consisted of 40% formamide, 1mM ribonucleoside vanadyl complex (Sigma-Aldrich), 10mM NaPO4, BSA (2mg/ml, Roche), and 1X SSC. Probe mixture for detection of C9 RNA foci consisted of 25 μL 80% formamide, 0.5 μL E. coli tRNA (20 μg μL, Thermo Fisher Scientific), 1 μL salmon sperm (10 μg/ μL, Thermo Fisher Scientific), and 0.4 μL 25μM 5′ digoxigenin (DIG)-labeled CCCCGGCCCCGGCCCC locked nucleic acid probe (Exiqon, batch 620574). Tris-buffered saline was comprised of 200mM Tris solution, pH 7.4, 50mM Tris and 15mM NaCl solution, pH 7.4 and 0.75% glycine. Blocking buffer consisted of 5% heat-shocked BSA in Tris-buffered saline and 10% normal donkey serum (Jackson Immunoresearch). Immunofluorescence buffer was comprised of 2% heat-shocked BSA in Tris-buffered saline. Detection of DIG-labeled probe was performed with a fluorescein-conjugated sheep anti-DIG antibody (1:250, Roche). All buffers were made with RNase-free PBS or water.

### Quantification of C9-GP DPR by ELISA

Cell pellets of *C9orf72* and isogenic control MNs co-cultured with murine astrocytes were collected and lysed in RIPA buffer (Thermo Fisher Scientific). Lysed material was sonicated for 5 pulses of 40Hz. Lysed material was centrifuged at 22,000 rpm for 15 minutes and supernatant was transferred to a new tube. The concentration of protein in the supernatant was quantified using the BCA protein assay (Thermo Fisher Scientific). 400μg of protein lysate was used for quantification of poly-GP. Lysates were loaded onto a 96-well plate (Meso Scale Discovery, Cat. no. L45XA) that had been coated with a custom-made polyclonal anti-(GP)_8_ antibody (1 μg/ml, Covance). Lysates were compared in multiple wells. Serial dilutions of recombinant (GP)8 peptide were prepared in 1% BSA+TBS-T for generation of the standard curve. For detection, anti-GP(8) tagged with GOLD SULFO (GOLD SULFO-TAG NHS-Ester Conjugation Pack, Meso Scale Discovery; Cat. no. R31AA) was used at a concentration of 0.5μg/mL. Quantification of signal from the assay plate was performed using a QuickPlex SQ120 instrument (Meso Scale Discovery). Sample groups were blinded prior to analysis.

### Immunocytochemistry

Fixation of cells was performed with 4% paraformaldehyde. Subsequently, cells were blocked in PBS with 0.2% Triton (Sigma-Aldrich) and 10% normal donkey serum (Jackson ImmunoResearch). Samples were incubated at 4°C overnight with primary antibodies in PBS with 5% wt/vol BSA. Following 3 washes with PBS + 0.1% Triton, samples were incubated at room temperature for 1 h with corresponding secondary antibodies conjugated to Alexa Fluor 488, Alexa Fluor 555, and Alexa Fluor 647. Finally, cell nuclei were stained with Hoechst 33342. Immunolabeled samples were imaged using a Nikon Ti2 Widefield and W1 spinning disk confocal photographed using a C10600-ORCA-R2 digital CCD camera (Hamamatsu Photonics, Japan).

### Image acquisition and analysis

Quantification of *SYN1*-GFP and propidium iodide was performed using ImageJ (U. S. National Institutes of Health, Bethesda, Maryland, USA) with the cell counter analysis plugin. In Figure 1, images were collected within 1.5 hours prior to trauma and then every 1.5 hours for the first 6 hours following trauma. Beyond this, images were collected every 12 hours for a total of 54 hours. For Figure 2, images were collected and *SYN1*-GFP quantified 1.5 hours prior to and 1.5 hours post-trauma. Images used for analysis in Figures 1 and 2 were obtained using the IncuCyte S3 live cell imager at 20X magnification with an exposure time of 300ms. Representative images shown in Supplementary Figure 2 were obtained using the Nikon Biostation. For Figure 3, quantitative analysis of RNA foci using FISH was performed on a Nikon W1 Spinning Disk Confocal using a 60x objective lens (slice depth 0.6μm). For Figures 4, 5, 7, and the accompanying Supplemental Figures analysis of TDP-43 staining was performed using images gathered from a Nikon Ti2 Widefield microscope at 40X magnification (slice depth = 1.2 μm; exposure times for DNA: 200ms, TDP-43: 800ms, MAP2: 2s). Representative images for TDP-43 localization analysis were obtained using a Nikon W1 Spinning Disk Confocal with a 100x silicone oil objective lens (slice depth = 0.4μm). For Figure 6, images of *SYN1*-GFP neurons were taken immediately prior to injury and at 6 and 12 days later at 20X with an exposure time of 300ms using the IncuCyte S3 live cell imager.

All experiments consisted of 3 independent differentiations, with at least 2 biological replicates per condition per experiment. Images for data analysis consisted of at least 9 randomly selected optical fields. For Figures 1, 2, and 6 *SYN1*-GFP was quantified by creating a threshold for *SYN1*-GFP positive neurons using Fiji. Figure 3 was analyzed using Nikon Elements software. Quantification of RNA foci was performed using a gain of 1.5 and background thresholded out on a per-slice basis. For Figures 4, 5, and 7 image analysis was performed using Nikon Elements analysis software. Analysis of nucleocytoplasmic ratios was performed using ROI tracing to compartmentalize the cytosol and nucleus. ROI analysis was used to determine the average (mean) fluorescence intensity across the region for both nuclear and cytosolic compartments, and the ratio represented was calculated by dividing the average nuclear fluorescence intensity by the average cytosolic fluorescence intensity. Images were analyzed and assembled as maximum projections from z-stack slices at the depths stated above. Representative images are presented with the same thresholding within each experiment. Images were assembled in Adobe Illustrator following direct export from Nikon Elements software. All experimental conditions were blinded to the operator prior to analysis.

### Statistical analysis

All statistical analysis was performed using GraphPad Prism 10 software. Experimental averages across differentiations for Figures 4C, 4E, 5B, 5D, 5F, 5H, 7C and 7E were standardized to 0 through z-score conversion. Data points were calculated relative to the experimental average and standard deviation based on the “no trauma” control condition of each respective experiment. Individual values were then standardized based on the degree of deviation from the experimental average relative to the experimental standard deviation.

Figure 1G: Survival analysis was performed using a two-way ANOVA with Tukey’s post-hoc multiple comparisons test indicating statistical significance between experimental group.

Figure 2C: qPCR analysis of ASO knockdown was performed in 3 biological replicates, each in triplicate. A mean of these 3 replicates is represented.

Figure 2D: analysis of *SYN1*-GFP quantification was performed using two-way ANOVA with Tukey’s post-hoc multiple comparisons tests indicating statistical significance between groups.

Figure 3: two-way ANOVA was performed for panels C, F, and G. Experimental averages from 3 independent experiments are represented for RNA foci. For DPR analysis, data are represented as the mean of 3 independent experiments performed concurrently. Tukey’s post-hoc multiple comparisons test indicates statistical significance between groups.

Figure 4C and 4D: statistical analysis was performed using a one-way ANOVA, with a Tukey’s post-hoc multiple comparisons test indicating statistical significance between groups.

Figure 5B, 5D, 5F, and 5H: statistical analysis was performed using a one-way ANOVA, with a Tukey’s post-hoc multiple comparisons test indicating statistical significance between groups.

Figure 5J and 5K: statistical analysis was performed using a Welch’s t-test.

Figure 6C and 6E: statistical analysis was performed using a two-way ANOVA, with a Tukey’s post-hoc multiple comparisons test indicates statistical significance between groups.

Figure 7C–F: statistical analysis was performed using a one-way ANOVA, with a Tukey’s post-hoc multiple comparisons test indicates statistical significance between groups.

## Supplementary Material

1
