# Supplementary material for: Traumatic injury causes selective degeneration and TDP-43 mislocalization in human iPSC-derived C9orf72-associated ALS/FTD motor neurons
Source: bioRxiv. 2024 Mar 26:2024.03.21.586073. Preprint. [Version 1] doi: 10.1101/2024.03.21.586073 (PMC10996466; doi:10.1101/2024.03.21.586073)
Supplement: 1 [file NIHPP2024.03.21.586073V1-supplement-1.pdf]

bioRxiv preprint doi: https://doi.org/10.1101/2024.03.21.586073; this version posted March 26, 2024. The copyright holder for this preprint (which was not certified by peer review) is the author/funder, who has granted bioRxiv a license to display the preprint in perpetuity. It is made available under aCC-BY 4.0 International license.

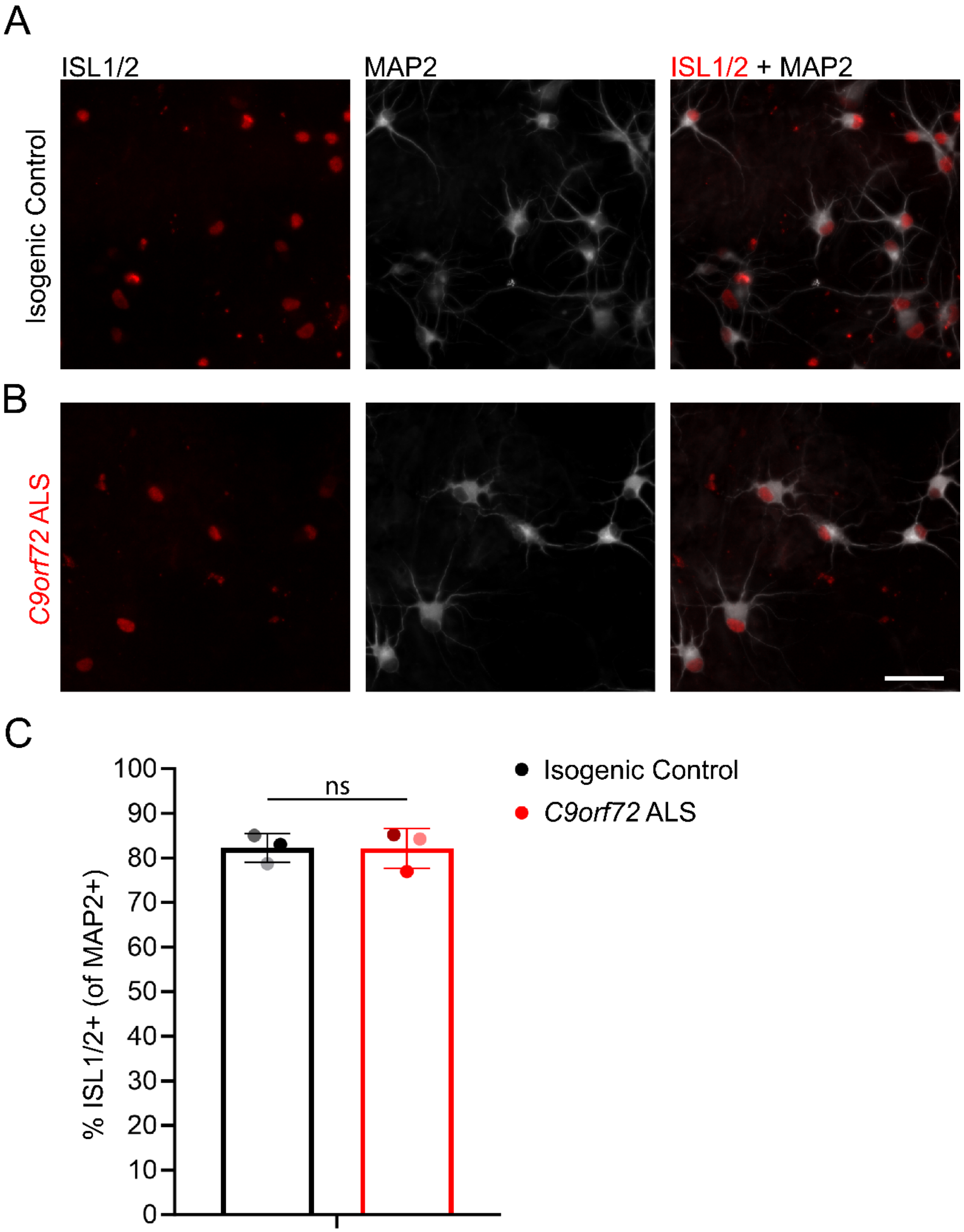

Supplementary Figure 1. Efficiency of differentiation in CS29 isogenic control and *C9orf72* ALS.

(**A**) Immunofluorescence depicting DNA, ISL1/2, and MAP2 in isogenic control and (**B**) *C9orf72* ALS. Scale bar = 50 μm.

(C) Graphical representation of experimental averages quantifying % ISL1/2+ motor neurons among MAP2+ neurons from 3 independent differentiations. Analysis performed with 24-28 fields of view quantifying 621-628 MAP2+ neurons. Mann Whitney p = NS.

bioRxiv preprint doi: https://doi.org/10.1101/2024.03.21.586073; this version posted March 26, 2024. The copyright holder for this preprint (which was not certified by peer review) is the author/funder, who has granted bioRxiv a license to display the preprint in perpetuity. It is made available under aCC-BY 4.0 International license.

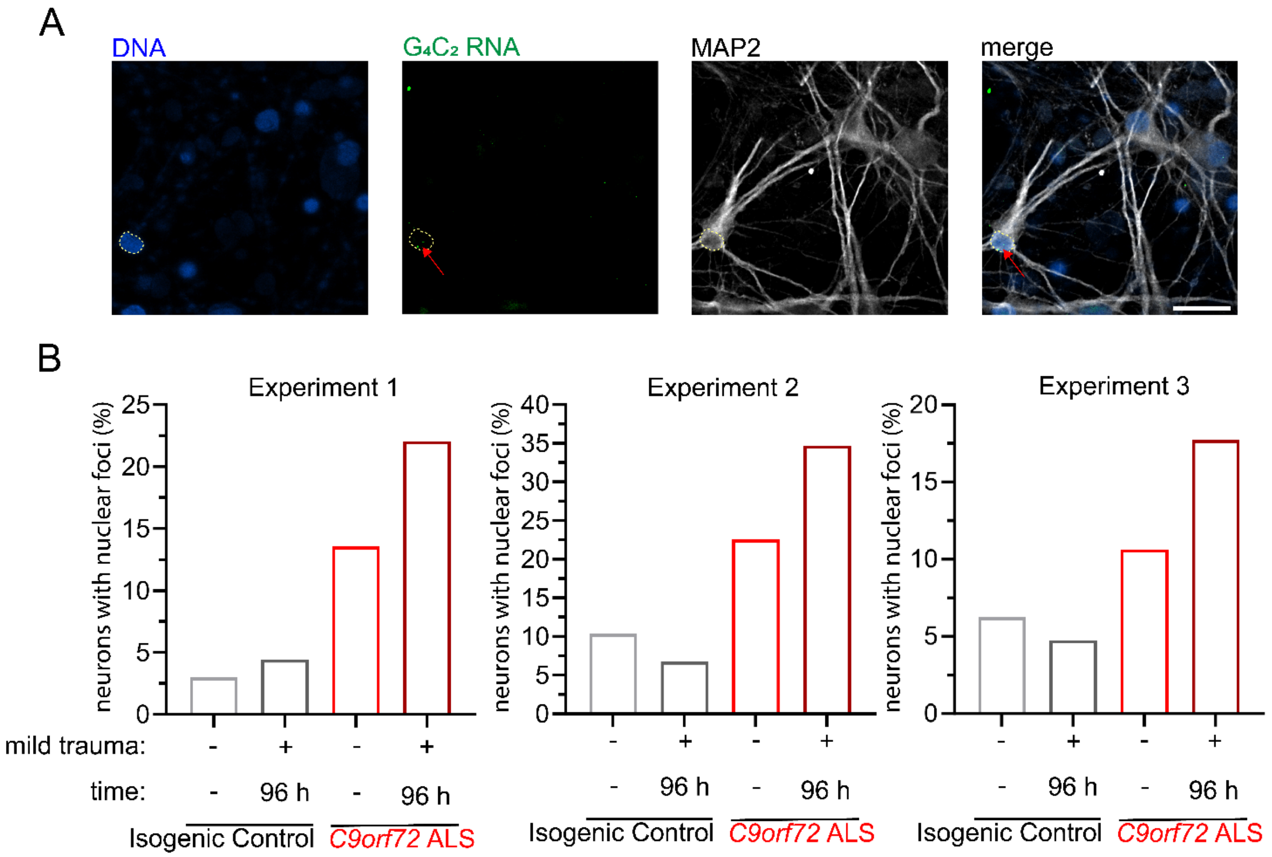

Supplementary Figure 2. Single channel characterization of nuclear G_4C_2 foci using FISH.

(A) Single channel zoomed out visualization of image used in Figure 3E. Red arrow indicates RNA foci and circle shows nuclear area. Scale bar = 25 µm.

(B) Experimental quantification of 3 independent differentiations noting the percentage of neurons with nuclear RNA foci.

bioRxiv preprint doi: https://doi.org/10.1101/2024.03.21.586073; this version posted March 26, 2024. The copyright holder for this preprint (which was not certified by peer review) is the author/funder, who has granted bioRxiv a license to display the preprint in perpetuity. It is made available under aCC-BY 4.0 International license.

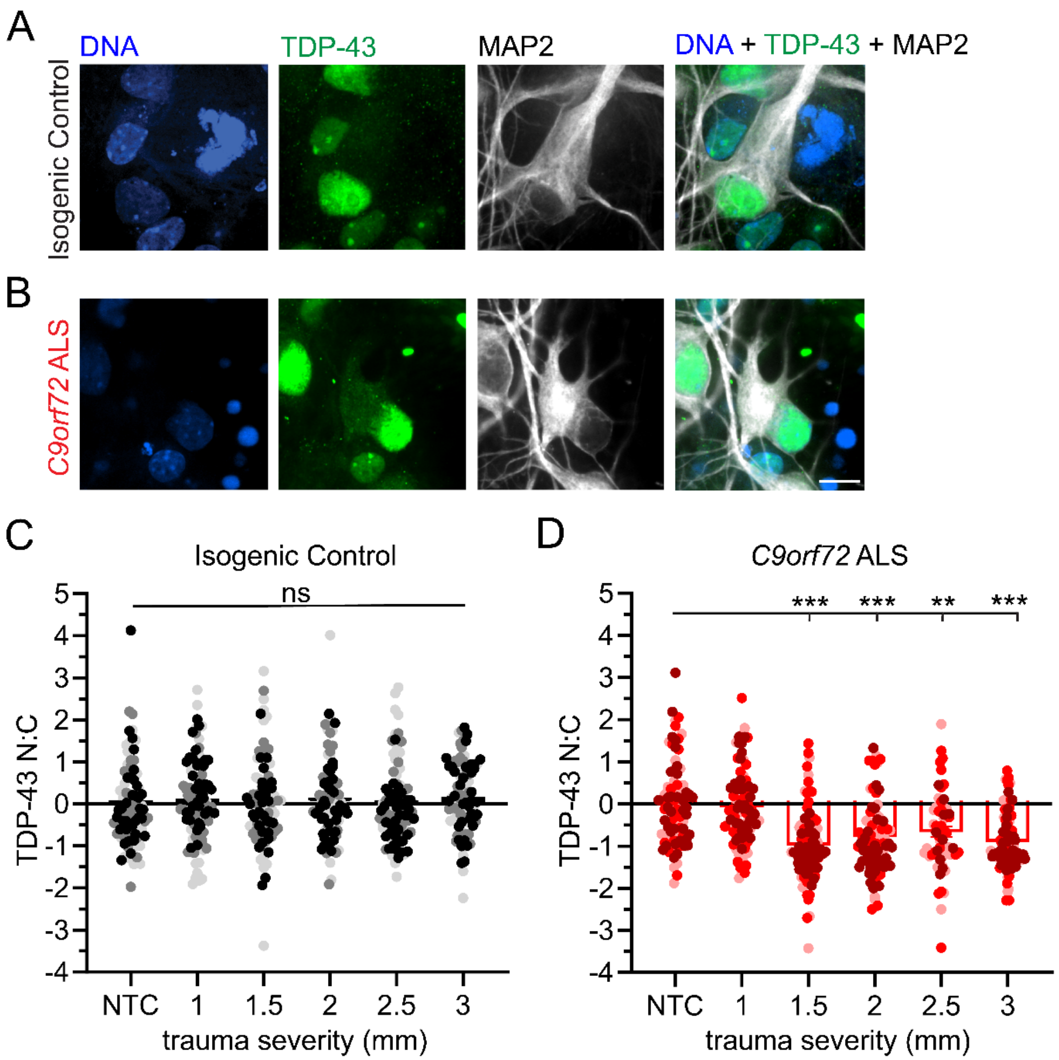

Supplementary Figure 3. Single channel characterization of TDP-43 staining 4 hours post-trauma.

(A-B) Single channel visualization of TDP-43 distribution in motor neurons in either isogenic control (**A**) or *C9orf72* ALS (**B**). Scale bar = 10 μm.

(C-D) Individual quantification of TDP-43 N/C across 3 independent differentiations in isogenic control (**C**) or *C9orf72* ALS (**D**) motor neurons. Different shades represent experimental distributions of individual cells. (**C**) Two-way ANOVA NS; (**D**) Two-way ANOVA $p < 0.0005$. Tukey's multiple comparisons test vs NTC. ** = $p < 0.01$, *** = $p < 0.005$.

bioRxiv preprint doi: https://doi.org/10.1101/2024.03.21.586073; this version posted March 26, 2024. The copyright holder for this preprint (which was not certified by peer review) is the author/funder, who has granted bioRxiv a license to display the preprint in perpetuity. It is made available under aCC-BY 4.0 International license.

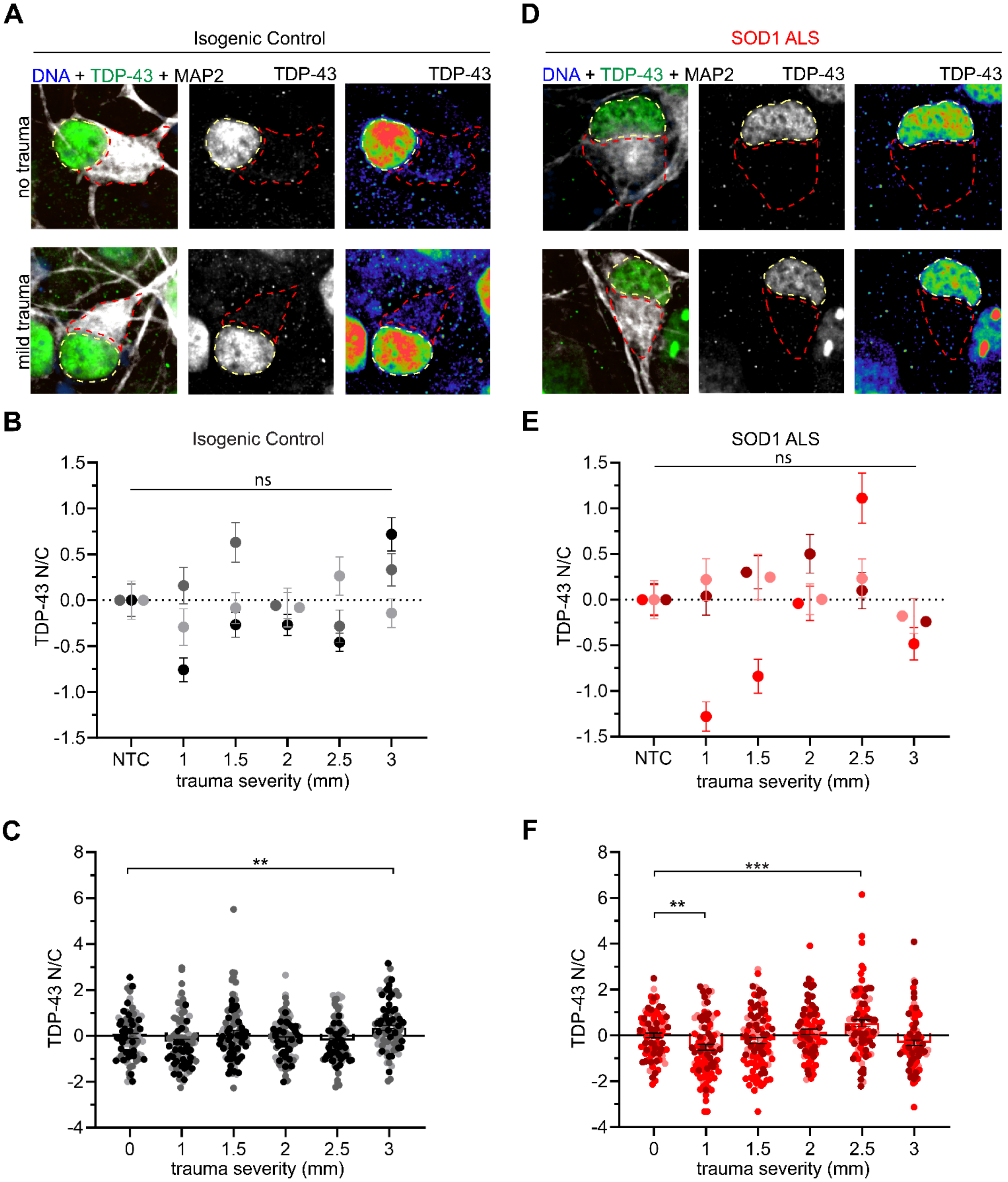

bioRxiv preprint doi: https://doi.org/10.1101/2024.03.21.586073; this version posted March 26, 2024. The copyright holder for this preprint (which was not certified by peer review) is the author/funder, who has granted bioRxiv a license to display the preprint in perpetuity. It is made available under aCC-BY 4.0 International license.

Supplementary Figure 4. SOD1 ALS motor neurons do not show cytosolic accumulation of TDP-43 following trauma.

(A and D) Immunocytochemistry showing TDP-43 distribution in motor neurons under no trauma and mild trauma conditions in isogenic control (**A**) and SOD1 ALS (**D**). Scale bar = 10 μm.

(B and E) Quantification of experimental averages from 3 independent differentiations of TDP-43 nucleocytoplasmic ratios in isogenic control (**C**) or SOD1 (**D**) motor neurons. One-way ANOVA, NS.

(C and F) Individual data points corresponding to one TDP-43 nucleocytoplasmic ratio per cell showing the overall data distribution in isogenic control (**C**) or SOD1 ALS (**F**) motor neurons. Analysis was performed on n = 87 - 104 cells per condition. One-way ANOVA, $p < 0.0001$. Tukey's multiple comparison test relative to NTC is indicated. ** = $p < 0.01$ *** = $p < 0.005$.

bioRxiv preprint doi: https://doi.org/10.1101/2024.03.21.586073; this version posted March 26, 2024. The copyright holder for this preprint (which was not certified by peer review) is the author/funder, who has granted bioRxiv a license to display the preprint in perpetuity. It is made available under aCC-BY 4.0 International license.

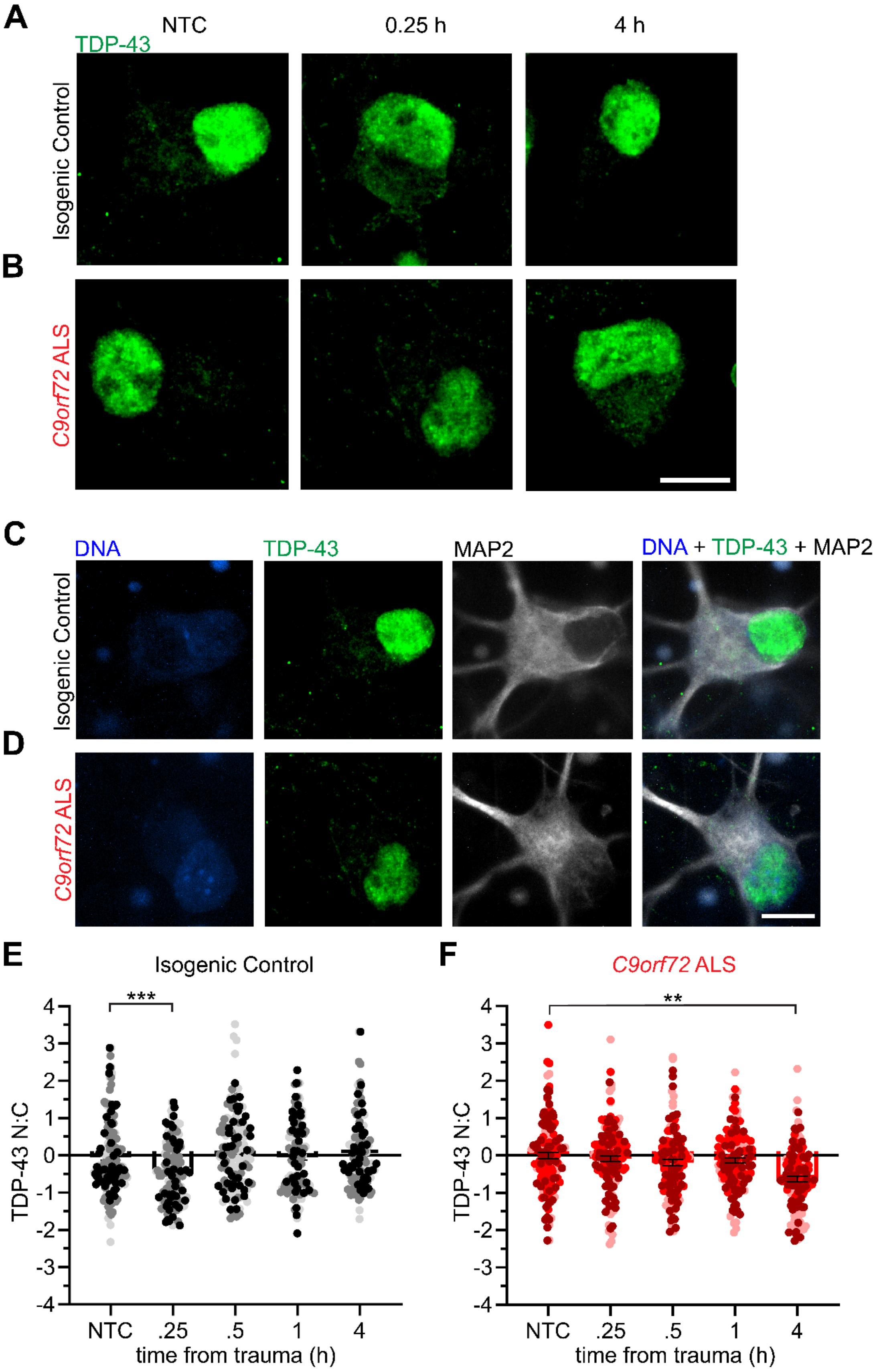

bioRxiv preprint doi: https://doi.org/10.1101/2024.03.21.586073; this version posted March 26, 2024. The copyright holder for this preprint (which was not certified by peer review) is the author/funder, who has granted bioRxiv a license to display the preprint in perpetuity. It is made available under aCC-BY 4.0 International license.

Supplementary Figure 5. Single channel characterization and individual data point analysis of motor neurons assessing immediate TDP-43 distribution following mild trauma.

(A-B) Visualization of representative GFP (TDP-43) staining without heatmap filter in isogenic control (**A**) or *C9orf72* ALS (**B**) motor neurons. Scale bar = 10 µm.

(C-D) Single channel visualization of representative images from either isogenic control (**C**) or *C9orf72* ALS (**D**) motor neurons. Scale bar = 10 µm.

(E-F) Data from individual cells used for quantification of experimental averages in Figure 5 for either isogenic control (**E**) or *C9orf72* ALS (**F**). Data are motor neurons from 3 independent differentiations. Different shades represent different experiments. n = 157-193. **(E)** One-way ANOVA, $p < 0.05$; **(F)** $p < .0001$; Tukey's multiple comparison test relative to NTC is indicated; ** = $p < 0.01$ *** = $p < 0.005$.

bioRxiv preprint doi: https://doi.org/10.1101/2024.03.21.586073; this version posted March 26, 2024. The copyright holder for this preprint (which was not certified by peer review) is the author/funder, who has granted bioRxiv a license to display the preprint in perpetuity. It is made available under aCC-BY 4.0 International license.

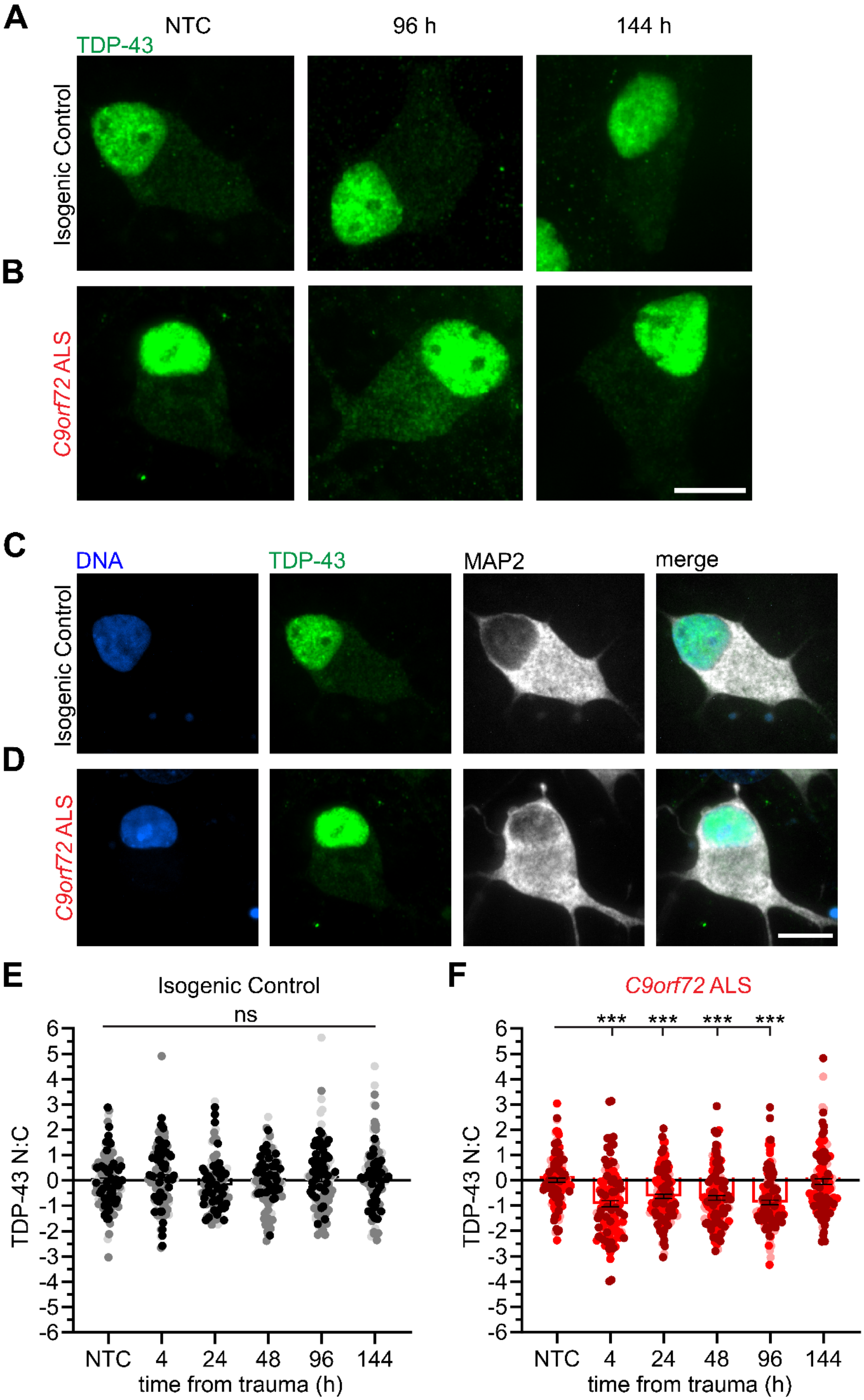

bioRxiv preprint doi: https://doi.org/10.1101/2024.03.21.586073; this version posted March 26, 2024. The copyright holder for this preprint (which was not certified by peer review) is the author/funder, who has granted bioRxiv a license to display the preprint in perpetuity. It is made available under aCC-BY 4.0 International license.

Supplementary Figure 6. Single channel characterization and individual data point analysis of motor neurons assessing extended TDP-43 distribution following mild trauma.

(A-B) Visualization of representative GFP (TDP-43) staining without heatmap filter in isogenic control (**A**) or *C9orf72* ALS (**B**) motor neurons. Scale bar = 10 μm.

(C-D) Single channel visualization of representative images from either isogenic control (**C**) or *C9orf72* ALS (**D**) motor neurons. Scale bar = 10 μm.

(E-F) Data from individual cells used for quantification of experimental averages in Figure 5 for either isogenic control (**E**) or *C9orf72* ALS (**F**). Data are motor neurons from 3 independent differentiations. Different shades represent different experiments. n = 124-133. One-way ANOVA, NS for **E**; $p < 0.0001$ for **F**. Tukey's multiple comparison test relative to NTC is indicated; *** = $p < 0.0005$.

bioRxiv preprint doi: https://doi.org/10.1101/2024.03.21.586073; this version posted March 26, 2024. The copyright holder for this preprint (which was not certified by peer review) is the author/funder, who has granted bioRxiv a license to display the preprint in perpetuity. It is made available under aCC-BY 4.0 International license.

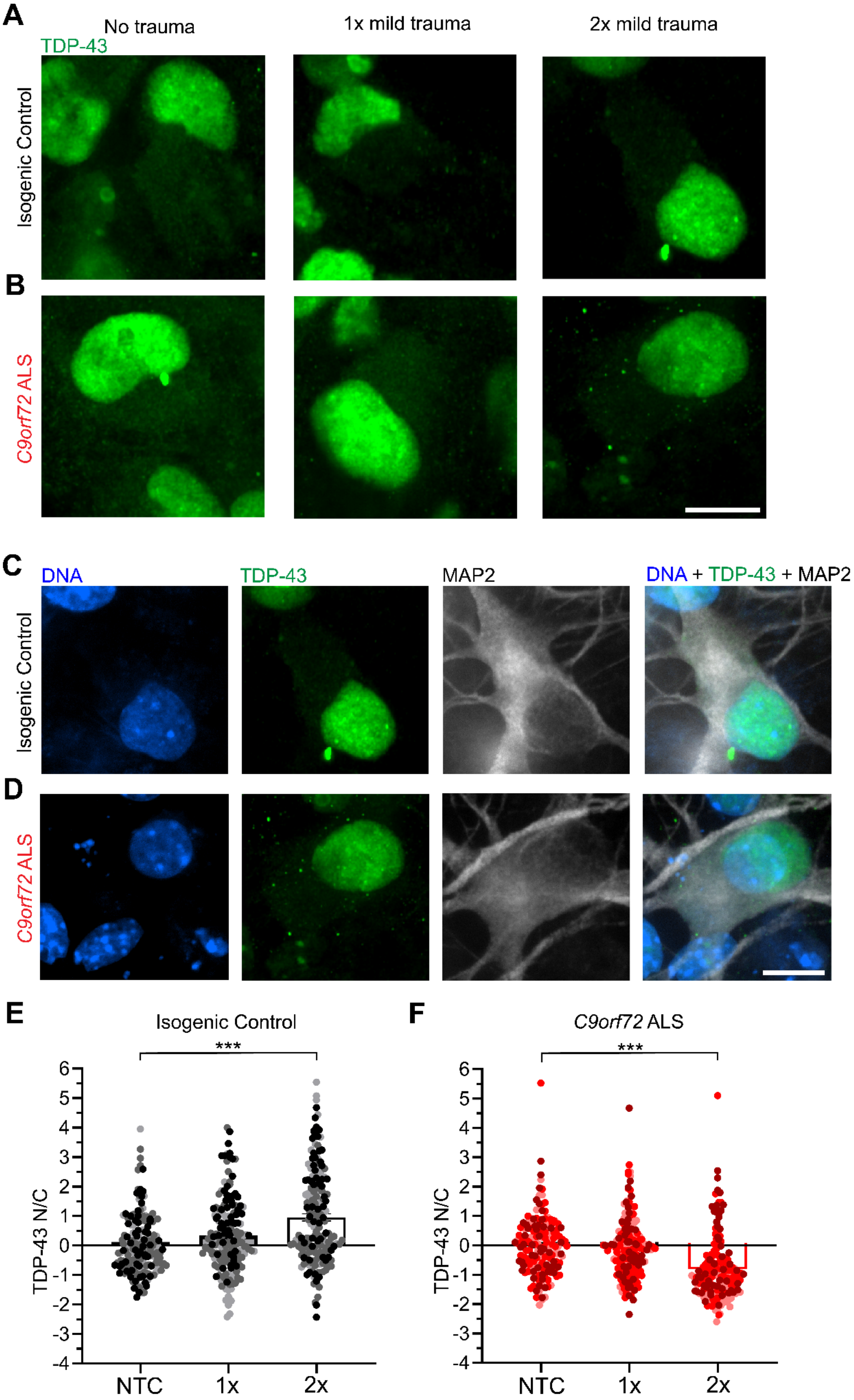

bioRxiv preprint doi: https://doi.org/10.1101/2024.03.21.586073; this version posted March 26, 2024. The copyright holder for this preprint (which was not certified by peer review) is the author/funder, who has granted bioRxiv a license to display the preprint in perpetuity. It is made available under aCC-BY 4.0 International license.

Supplementary Figure 7. Single channel characterization and individual data point analysis of motor neurons assessing TDP-43 distribution following repetitive mild trauma.

(A-B) Visualization of TDP-43 distribution (GFP) without heatmap filter in isogenic control (**A**) or *C9orf72* ALS **(B)** motor neurons. Scale bar = 10 μm.

(C-D) Single channel image visualization of representative images for isogenic control (**C**) or *C9orf72* ALS (**D**) motor neurons. Scale bar = 10 μm.

(E-F) Individual data points for experimental averages used in Figure 7 in either isogenic control (**E**) or *C9orf72* ALS (**F**). Data are motor neurons from 3 indpeendent differentiations. Different shades represent different experiments. n = 168-179. Two-way ANOVA for **E** and **F** $p < 0.0001$. Tukey's multiple comparison test relative to NTC is indicated. *** = $p < 0.005$.

SUPPLEMENTARY VIDEOS

Supplementary Video 1. High magnification visualization of stretch trauma. Increased resolution emphasizing vertical and horizontal stretch dynamics of the post array.

Supplementary Video 2: Induced stretch trauma of a flexible, PDMS-bottomed plate. Video shows 30FPS high-speed camera capture of a spraypainted wells. The left well is a no-trauma control, while the right well depicts a 2mm displacement. The second video depicts a higher resolution video of the stretched well.
